# Effect of antidepressants for cessation therapy in betel-quid use disorder: a randomised, double-blind, placebo-controlled trial

**DOI:** 10.1017/S2045796020000384

**Published:** 2020-05-06

**Authors:** Chung-Chieh Hung, Chien-Hung Lee, Albert Min-Shan Ko, Hsien-Yuan Lane, Chi-Pin Lee, Ying-Chin Ko

**Affiliations:** 1 Graduate Institute of Clinical Medical Science, China Medical University, Taichung, Taiwan; 2 Department of Psychiatry, China Medical University Hospital, Taichung, Taiwan; 3 Department of Public Health and Research Center for Environment Medicine, Kaohsiung Medical University, Kaohsiung, Taiwan; 4 Department of Medical Research, Kaohsiung Medical University Hospital, Kaohsiung, Taiwan; 5 Key Laboratory of Vertebrate Evolution and Human Origins of Chinese Academy of Sciences, Institute of Vertebrate Paleontology and Paleoanthropology, Chinese Academy of Sciences, Beijing, China; 6 Department of Psychology, College of Medical and Health Sciences, Asia University, Taichung, Taiwan; 7 Environment-Omics-Disease Research Center, China Medical University Hospital, China Medical University, Taichung, Taiwan

**Keywords:** Clinical drug studies, common mental disorders, epidemiology, psychoactive substance use disorder, randomised controlled trials

## Abstract

**Aims:**

More than one-half of betel-quid (BQ) chewers have betel-quid use disorder (BUD). However, no medication has been approved. We performed a randomised clinical trial to test the efficacy of taking escitalopram and moclobemide antidepressants on betel-quid chewing cessation (BQ-CC) treatment.

**Methods:**

We enrolled 111 eligible male BUD patients. They were double-blinded, placebo-controlled and randomised into three treatment groups: escitalopram 10 mg/tab daily, moclobemide 150 mg/tab daily and placebo. Patients were followed-up every 2 weeks and the length of the trial was 8 weeks. The primary outcome was BQ-CC, defined as BUD patients who continuously stopped BQ use for ⩾6 weeks. The secondary outcomes were the frequency and amount of BQ intake, and two psychological rating scales. Several clinical adverse effects were measured during the 8-week treatment.

**Results:**

Intention-to-treat analysis shows that after 8 weeks, two (5.4%), 13 (34.2%) and 12 (33.3%) of BUD patients continuously quit BQ chewing for ⩾6 weeks among placebo, escitalopram, moclobemide groups, respectively. The adjusted proportion ratio of BQ-CC was 6.3 (95% CI 1.5–26.1) and 6.8 (95% CI 1.6–28.0) for BUD patients who used escitalopram and moclobemide, respectively, as compared with those who used placebo. BUD patients with escitalopram and moclobemide treatments both exhibited a significantly lower frequency and amount of BQ intake at the 8th week than those with placebo.

**Conclusions:**

Prescribing a fixed dose of moclobemide and escitalopram to BUD patients over 8 weeks demonstrated treatment benefits to BQ-CC. Given a relatively small sample, this study provides preliminary evidence and requires replication in larger trials.

## Introduction

Approximately 600 million people chew betel-quid (BQ) worldwide, making it the fourth most popular accepted psychoactive substance used in daily life (Gupta and Warnakulasuriya, 2002). It is used for social bonding and cultural practice among individuals in India, Taiwan, Southern China, Southeast Asia, South Pacific countries and migrant communities in the UK and USA (Osborne *et al*., 2017). BQ is a chewing mixture of dried or fresh substance from the *Areca catechu* nut (AN) that is taken with or without tobacco (Lee *et al*., 2011, 2014). In 2004, BQ and AN taken with or without tobacco are both listed as a human Group 1 carcinogens by the International Agency for Research on Cancer and closely linked to the risk of contracting upper aerodigestive tract cancers (IARC, 2004; Lee *et al*., 2012*b*).

In 2018, Lee *et al.* validated BQ use disorder (BUD) among addictive BQ users using *DSM-5* diagnosis of ‘use disorder’ in six BQ endemic Asian populations (Lee *et al*., 2018). For Taiwan, Mainland China, Malaysia, Indonesia, Nepal and Sri Lanka, the 12-month prevalence of *DSM-5*-defined BUD were determined to be 56−99% of current users (Lee *et al*., 2012*a*, 2018). Furthermore, studies are confirming that BQ dependence is organic brain dysfunction, in that neuroimaging alterations in BQ-dependent users can be observed on functional magnetic resonance imaging (Liu *et al*., 2016*a*, 2016*c*). The physiological and pathological changes are identified in the brain, predominantly in the activation of reward system that increases functional connectivity from anterior cingulate cortex to the regions of the reward network (Liu *et al*., 2016*b*; Huang *et al*., 2017).

Previous findings from our laboratory have shown that certain monoamine oxidase A (MAOA) gene variants are associated with heavy BQ users (Chen *et al*., 2012). Cell and animal models revealed that AN and arecoline inhibited MAOA mRNA and protein expression, as well as having some monoamine oxidase inhibitor (MAOI) like properties (Dar and Khatoon, 2000; Chen *et al*., 2012). The MAO catalyses the deamination in the synapses and regulates the levels of dopamine, serotonin, norepinephrine and catecholamine, and its inhibition similarly works as an antidepressant (Fowler and Tipton, 1984). Thus, the use of MAOI may have a clinical effect for BQ cessation among heavy BQ users. Selective serotonin reuptake inhibitors (SSRI) can constrain the serotonin re-uptake transporter to enhance the serotonergic neuro-transmission in the synapses, and are considered a better tolerated antidepressant, as compared to MAOI (Raymond *et al*., 2001). Because SSRI can increase the level of serotonin in the brain, the use of SSRI is also presumed to have a treatment effect for BQ cessation among heavy BQ chewers.

To date, no pharmacologically based cessation therapy is available to assist with alleviating the BUD patients who want to reduce or quit BQ use. Government regulation or counselling-based cessation programmes have been the only avenues for BQ users (Le *et al*., 2014; Osborne *et al*., 2017). However, previous findings and statistics have shown limited efficacy of these quitting programmes (Le *et al*., 2014). Thus, the development of a pharmacologically assistant therapy for BQ dependence may provide a drug-based approach for BQ chewing cessation (BQ-CC) therapy (Osborne *et al*., 2017). The aims of this clinical trial were to test: (1) Can prescribing escitalopram (SSRI) and moclobemide (MAOI) increase BQ quit rates among BUD patients as compared to prescribing placebo? (2) Can escitalopram and moclobemide treatments decrease the frequency and amount of BQ intake among BUD patients as compared to placebo treatment?

## Methods

### Design

A double-blinded, placebo-controlled and randomised clinical trial was conducted to evaluate the study. We recruited BQ participants from the cancer-screening centre or those referred from the department of dentist and general physicians at China Medical University Hospital, Taichung, Taiwan. BQ users who had an intention to participate in this study were requested to achieve several psychological assessments for participant inclusion and exclusion criteria at the Department of Psychiatry in the study hospital.

First, the BUD status was evaluated using a standardised clinical interview by a psychiatrist. The 11 symptoms of *DSM-5* criteria were employed to diagnose BUD (Lee *et al*., 2018). The symptoms were assessed as: (1) large amount or longer history of BQ use; (2) unsuccessful cut down; (3) time spent chewing; (4) craving; (5) neglected major roles; (6) social or interpersonal problems; (7) given up activities; (8) hazardous use; (9) continued use despite knowing problems; (10) tolerance; and (11) withdrawal. BUD was defined as having ⩾2 of the above symptoms in the past one year. Of those, BQ users with 2−3 symptoms were defined as having mild BUD, those with 4−5 symptoms as having moderate BUD, and those with ⩾6 symptoms as having severe BUD. Second, we assessed several psychiatric disorders using *DSM-5* diagnostic methods, and investigated the antidepressants used for this disorder. The disorders included depressive disorders, opioid and opioid-like substance use disorders diagnosed via the semi-structured diagnostic interview carried out by one study psychiatrist (Smaga *et al*., 2017; Shankman *et al*., 2018). The diagnosis of cancer and pre-cancer was determined in the cancer-screening centre of the study hospital. Neurological diseases or other organic brain disorders were determined according to the patients' self-reports on their lifetime medical histories.

### Participants

The lifetime prevalence of BQ use among women in Taiwan was extremely low, at ~3.0% of BQ users (Lee *et al*., 2011), thus male BQ users were the recruitment targets for this clinical trial. In Taiwan, all BQ products are without tobacco and no BQ users are tobacco chewers (Lee *et al*., 2011). The inclusion criteria for this study are as follows: (1) male BQ users aged 18–65 years who have used BQ for at least one year; (2) satisfied criteria for BUD diagnosis; (3) who is fluent in Chinese or Taiwanese language. Subjects diagnosed with the following disorders or diseases were excluded from our study: (1) *DSM-5*-defined depressive disorders and undergoing traditional antidepressants (e.g. SSRIs, TCAs and MAOIs). Except for hypnotics, participants who used antipsychotics, mood stabilisers or other medication that can influence the central nervous system were also excluded; (2) opioid and opioid-like substance use disorders in the past one year; (3) other substance use disorders in the past one year, except alcohol, caffeine and nicotine; (4) cancer and pre-cancer diseases; (5) neurological diseases or other organic brain disorders, such as intellectual disability and stroke.

A total of 139 male current BUD patients were determined to be eligible between 5 January 2016 and 19 March 2018. Of those, 28 refused to participate or were not contactable later. This clinical trial was approved by the China Medical University and Hospital Research Ethics Committee. All participants gave written informed consent. This clinical trial has been registered in a public database for clinical studies (ClinicalTrials.gov, ID: NCT 03010761).

### Randomisation and masking

Online Supplementary Fig. S1 shows the participant flowchart for the randomised clinical trial. A total of eligible 111 male patients with BUD were randomised into one of the three drug treatment groups: placebo (artificial capsules with no therapeutic or pharmacological effects, *n* = 37 cases), escitalopram (SSRI 10 mg/tab daily, *n* = 38 cases) and moclobemide (MAOI 150 mg/tab daily, *n* = 36 cases). In this study, a fixed dosage of 10 mg/day of escitalopram and 150 mg/day of moclobemide were used, respectively. Shen *et al*. (2019) described the range of effective dosages for the treatment of depression as being 10–20 mg/day of escitalopram and Bonnet (2003) described the range of 300–600 mg/day (divided into 2–3 doses) for 4–6 weeks of treatment (Bonnet, 2003; Shen *et al*., 2019). Because our participants were not depressed, we used the lower dosage (i.e. 10 mg/day) for escitalopram and one-half dosage of 300 mg/day (i.e. 150 mg/day) to examine their effects on this substance use disorder. The above trial drugs were inserted into one capsule with the same appearances. The China Chemical & Pharmaceutical Co., Ltd. offered us all of the treatment drugs (Taipei City, Taiwan, 2017).

All participants were regularly evaluated for the BQ use- and disorder-related outcomes at 2-, 4-, 6- and 8-week follow-up after drug treatment in accordance with double-blind procedures. Twenty-six (70.3%), 34 (89.5%) and 31 (86.1%) participants completed 8-week follow-up in these three treatment groups, respectively. Detail follow-up rates for all follow-up points were shown in Table 1. Reasons for data with missing included failure to return the follow-up, refusing the continuous treatments and insufficient responses (overall, nine, nine and two users, respectively).

**Table 1.** Distributions of baseline characteristics among male betel-quid use disorder chewers, stratified by drug treatment groups

Factors^a^	Placebo	Escitalopram	Moclobemide
	*n* = 37	*n* = 38	*n* = 36
*Demographic factors*			
Age, years	41.9 ± 10.8	40.9 ± 7.5	41.2 ± 10.2
Educational level, years	10.5 ± 2.4	11.4 ± 2.4	10.3 ± 3.5
Cigarette smoking	100.0%	100.0%	94.4%
*Characteristics of BQ use*			
Starting age, years	17.8 ± 5.2	16.4 ± 5.8	16.8 ± 4.1
Frequency, days/week	6.2 ± 1.8	5.3 ± 2.3	6.3 ± 1.7
Amount, quids/day	28.4 ± 35.2	40.0 ± 48.9	50.5 ± 48.1
Types			
Areca nut with betel leaf	73.0%	73.7%	72.2%
Areca nut with betel inflorescence	21.6%	15.8%	25.0%
Mixed use	5.4%	10.5%	2.8%
*BQ use disorder*			
No. of *DSM-5* symptom	7.1 ± 2.9	7.1 ± 2.6	7.4 ± 2.7
*DSM-5*-defined BUD			
Mild	16.2%	7.9%	11.1%
Moderate	13.5%	23.7%	11.1%
Severe	70.3%	68.4%	77.8%
*Psychological rating scale, point*			
SUSRS	12.5 ± 4.8	13.2 ± 4.9	13.9 ± 4.7
YB-OCDRS	28.1 ± 9.2	29.7 ± 10.2	29.9 ± 8.8
*Other substance use disorder, point*			
*DSM*-5 criteria for cigarette use	6.9 ± 3.2	7.4 ± 3.8	7.4 ± 3.1
SUSRS for cigarettes use	13.2 ± 5.6	13.1 ± 4.8	13.5 ± 5.5
*DSM*-5 criteria for alcohol use	3.2 ± 4.5	3.4 ± 4.7	3.8 ± 4.4
SUSRS for alcohol use	5.8 ± 7.5	6.7 ± 7.6	7.2 ± 7.8
*Psychiatric status, point*			
HDRS	5.6 ± 6.3	4.5 ± 5.3	5.7 ± 7.0
BAI	16.4 ± 16.9	14.2 ± 13.7	12.3 ± 12.5
BDI	14.5 ± 13.5	11.3 ± 14.6	15.4 ± 15.8
*Cigarette smoking, yes*	100.0%	100.0%	94.4%
*Follow-up information*			
Total follow-up time, week	6.4 ± 2.6	7.4 ± 1.9	7.2 ± 2.1
Completing 2-wks follow-up	81.1%	92.1%	86.1%
Completing 4-wks follow-up	78.4%	89.5%	86.1%
Completing 6-wks follow-up	70.3%	89.5%	86.1%
Completing 8-wks follow-up	70.3%	89.5%	86.1%
Completing BQ-CC assessment	75.7%	92.1%	86.1%

BQ, betel-quid; BUD, BQ use disorder; *DSM-5*, the 5th edition of Diagnostic and Statistical Manual of Mental Disorders; SUSRS, Substance Use Severity Rating Scale; YB-OCDRS, Yale-Brown Obsessive Compulsive Disorder Rating Scale; HDRS, Hamilton Depression Rating Scale; BAI, Beck anxiety inventory; BDI, Beck depression inventory; BQ-CC, BQ chewing cessation.

a All data are presented in mean ± standard deviation.

### Procedures

The research team included one full-time psychiatrist, two study nurses, several researchers and a principal investigator. The participants, psychiatrist, nurses and researchers all were masked to assignments for study interventions. Participants were randomly allocated to receive the escitalopram, moclobemide or placebo treatments with the ratio of 1:1:1 according to a block randomisation scheme. This method is commonly used in clinical trials to decrease bias and attain equilibrium in the allocation of participants to treatment arms, particularly for studies with small sample size (Efird, 2011). The sequence of drug treatment allocation was concealed in opaque, security-sealed envelopes, held centrally until randomisation code release by the third party. The principal investigator and a biostatistical expert generated the random allocation sequence. Two nurses were trained as the case managers to enrol study participants. All participants were assigned to treatment arms by the psychiatrist according to a pre-determined sequence. Study nurses obtained information on age at which BQ users started using BQ, usage frequency, the amount of daily intake, as well as the consumption of alcohol and tobacco at baseline and each follow-up evaluation. To monitor the adherence to drug protocol, the study nurses daily checked the condition of drug use via telephone interview to each participant. All the baseline and follow-up interviews were administered by the team principal investigator. Because the principal aim of this clinical trial was to investigate the effect of medication on the cessation of BQ users, we did not offer any type of counselling to the participants.

### Measures

At each follow-up evaluation, participants were requested to complete a checklist for the days taking their medicine and if there were any discomforts after taking the drug treatment. An independent psychiatrist, who was unaware of the drug treatment for each participant and did not have access to any supervision notes, performed the outcome evaluations.

The Substance Use Severity Rating Scale (SUSRS) was developed by *DSM-IV* and ICD-11 diagnostic systems (First *et al*., 2005; Smaga *et al*., 2017). This scale contains 21 different items for measuring the severity of addictiveness of substance use for patients with clinical disorder or dependence, and has been widely used for cigarette and drug use (Tsai *et al*., 2012; Mercincavage *et al*., 2016). At each follow-up point, we measured participants' SUSRS scores for alcohol, cigarettes and BQ. The rater checked the participants for the using condition of these substances, recoding ‘yes or no’ as score ‘1 or 0’ in the scales every 2 weeks in the overall 8 weeks. We also checked the participants' score on the Hamilton Depression Rating Scale (Hamilton, 1960). Self-report Beck anxiety inventory and Beck depression inventory were also used during each follow-up (Beck *et al*., 1988, 1996). We used the Yale-Brown Obsessive Compulsive Disorder Rating Scale (YB-OCDRS) to measure the behavioural problems of BQ patients (Goodman *et al*., 1989*a*, 1989*b*). YB-OCDRS is a tool commonly employed to examine the severity of craving or desire for substance use (Connor *et al*., 2005). We translated YB-OCDRS into the Chinese language for BQ addictive use. A previous study demonstrated that this scale is a reliable and valid instrument for assessing the carving of alcohol use in Chinese populations (Gau *et al*., 2005).

The primary outcome was BQ-CC that is defined as BUD patients who continuously stopped BQ use for ⩾6 weeks. The status of chewing cessation was verified using the level of arecoline (the major metabolite of areca nut) in the urine samples that were collected at the 8th week of follow-up. The automated online SPE LC-MS/MS method was used to determine the urinary level of arecoline (Hu *et al*., 2010). The level of arecoline in BQ cessation individuals (mean ± s.d.: overall, 14.6 ± 45.0; placebo, 1.9 ± 2.7; escitalopram, 15.7 ± 47.1; moclobemide, 15.5 ± 48.3 ng/ml) was significantly lower than that for non-cessation individuals (mean ± s.d.: overall, 178.5 ± 347.8; placebo, 192.3 ± 391.7; escitalopram, 209.9 ± 393.5; moclobemide, 126.2 ± 225.9 ng/ml; *p* for the overall difference is 0.0016, obtained from the Wilcoxon rank-sum test). Also, following the smoking cessation guidelines, we classified BQ chewers who did not complete the assessment at 8 weeks as continuing to chew BQ. The secondary outcomes were continuous SUSRS and YB-OCDRS rating scores and the frequency and amount of BQ consumed measured at each follow-up.

### Safety

At each follow-up point, participants were carefully interviewed by the study nurses and were requested to fill in a checklist for their adverse events or side effects during the treatments. The checklist is a short form of structured UKU side effect rating scale, a kind of comprehensive and sensitive scale for the side effects related to the psychotropic medication use (Lingjaerde *et al*., 1987). The types of adverse events that were monitored included psychic (e.g. depression, concentration difficulty and failing memory), neurological (e.g. tremor, rigidity and epileptic seizure) and autonomic (e.g. nausea, diarrhoea and tachycardia) side effects, and others. In addition, biochemical examinations, including the levels of glutamic oxaloacetic transaminase, glutamic pyruvic transaminase, blood urea nitrogen, creatinine, sodium, potassium, white blood cell, haemoglobin and platelets were performed before and after 8-week drug treatments for all participants.

### Sample size and statistical power

In the stage of study design, we used binary BQ-CC as the primary outcome in the 8 weeks of follow-up. Initially, we planned to use the survival method to analyse our data. Given a two-tailed Cox proportional model with a type I error of 0.05, 80% of power and a 2.0 hazard ratio (twofold likelihood of BQ-CC for drug treatments of escitalopram or moclobemide as compared to placebo), a 37, 37 and 37 estimated sample for each treatment group was obtained. In the stage of the research end, we used our study findings and recruited participants to check for statistical power. Given 37, 38 and 36 samples for placebo, escitalopram and moclobemide, respectively, with a 5.4% of BQ-CC proportion for placebo, type I error of 0.05, an observed risk ratio (proportion ratio) of 6.3 and 6.8 for escitalopram and moclobemide, respectively, as compared to placebo (data in Table 3), the statistical power was calculated by a two-tailed likelihood-ratio test to be 0.899 and 0.933.

### Statistical analysis

All data analyses have complied with intention-to-treat procedures whereby participants were analysed according to the randomisation scheme (Liao *et al*., 2017), and their initial treatment assignment by Stata version 15 (StataCorp, USA). We employed multivariable logistic regression model to estimate proportion difference (measured in risk difference) and proportion ratio (measured in risk ratio) of BQ-CC between escitalopram and placebo treatments, and between moclobemide and placebo treatments (Norton *et al*., 2001). To diminish the residual confounding effect, all multivariable models were adjusted for age, education, cigarette smoking and the level of BUD. We used generalised estimating equations (GEE) with an autoregressive correlation structure to assess the interaction effect of drug type and treatment time on continuous secondary outcomes over 8-week follow-up (Zeger and Liang, 1986). GEE is developed to evaluate longitudinally repeated measurement data with missing either in specific follow-up sessions or withdrawal.

## Results

### Baseline and follow-up distributions

Among the 111 male patients, mean (s.d.) age was 41.3 ± 9.5 years. Table 1 shows the baseline characteristics of participants randomly allocated to placebo, escitalopram and moclobemide treatments. The proportion of severe BUD in placebo, escitalopram and moclobemide groups was 70.3, 68.4 and 77.8%, respectively. All three groups of BUD participants showed a comparable proportion of completing the 2-, 4-, 6- and 8-week follow-ups, and the BQ-CC assessment.

The distributions of BQ use characteristics, disorder rating scales and psychiatric status at baseline and 8 weeks of follow-up among BUD chewers with different drug treatments were shown in Table 2. The frequency and amount of BQ intake among participants treated with escitalopram and moclobemide were found to be decreased with increased treatment time. Similar trends in SUSRS and YB-OCDRS rating scales were observed in moclobemide users.

**Table 2.** Distributions of betel-quid use characteristics, disorder rating scales and psychiatric status at baseline and 8 weeks of follow-up among male BUD chewers with drug treatments

Factors^a^	Baseline	Week 2	Week 4	Week 6	Week 8
	*n* = 111	*n* = 96	*n* = 94	*n* = 91	*n* = 91
*Characteristics of BQ use*					
Frequency, days/week					
Placebo	6.2 ± 1.8	4.9 ± 2.7	5.0 ± 2.7	5.2 ± 2.5	5.5 ± 2.5
Escitalopram	5.3 ± 2.3	4.7 ± 3.0	4.0 ± 3.0	3.6 ± 3.2	3.2 ± 3.2
Moclobemide	6.3 ± 1.7	3.8 ± 3.1	3.1 ± 3.2	2.8 ± 2.9	2.5 ± 2.9
Amount, quids/day					
Placebo	28.4 ± 35.2	18.7 ± 21.2	21.9 ± 21.8	22.8 ± 22.5	33.1 ± 38.2
Escitalopram	40.0 ± 48.9	20.6 ± 29.5	16.0 ± 16.6	14.9 ± 20.7	13.0 ± 20.1
Moclobemide	50.5 ± 48.1	27.1 ± 44.6	21.5 ± 32.6	14.9 ± 20.5	11.4 ± 19.0
*BQ use disorder*					
SUSRS, point					
Placebo	12.5 ± 4.8	10.8 ± 6.6	9.2 ± 6.5	9.2 ± 8.2	10.2 ± 8.8
Escitalopram	13.2 ± 4.9	11.7 ± 7.2	9.5 ± 7.4	10.1 ± 8.2	8.6 ± 8.5
Moclobemide	13.9 ± 4.7	10.3 ± 7.1	8.6 ± 8.5	7.6 ± 6.8	5.5 ± 5.9
YB-OCDRS, point					
Placebo	28.1 ± 9.2	25.0 ± 12.0	22.0 ± 13.7	22.6 ± 17.2	22.5 ± 17.1
Escitalopram	29.7 ± 10.2	25.7 ± 13.7	20.8 ± 14.8	22.6 ± 16.7	20.0 ± 17.9
Moclobemide	29.9 ± 8.8	22.9 ± 14.0	20.7 ± 16.9	18.3 ± 14.1	14.3 ± 12.5
*Psychiatric status*					
HDRS, point					
Placebo	5.6 ± 6.3	4.3 ± 5.9	4.2 ± 6.0	3.7 ± 8.5	3.2 ± 6.8
Escitalopram	4.5 ± 5.3	2.4 ± 3.8	3.1 ± 4.8	3.7 ± 6.2	2.6 ± 5.3
Moclobemide	5.7 ± 7.0	3.7 ± 5.3	5.3 ± 10.8	3.5 ± 6.5	2.6 ± 6.0
BAI, point					
Placebo	16.4 ± 16.9	9.7 ± 13.7	8.5 ± 12.6	6.3 ± 10.6	7.9 ± 14.7
Escitalopram	14.2 ± 13.7	9.1 ± 12.2	8.1 ± 12.4	7.4 ± 11.0	5.9 ± 9.5
Moclobemide	12.3 ± 12.5	6.8 ± 10.9	7.6 ± 12.9	6.1 ± 13.2	6.9 ± 14.8
BDI, point					
Placebo	14.5 ± 13.5	13.0 ± 17.1	10.4 ± 14.7	6.7 ± 10.7	8.3 ± 14.8
Escitalopram	11.3 ± 14.6	8.9 ± 12.7	9.9 ± 14.6	9.2 ± 13.6	8.6 ± 13.2
Moclobemide	15.4 ± 15.8	7.7 ± 9.6	11.0 ± 15.7	7.3 ± 13.7	8.0 ± 18.2

BQ, betel-quid; BUD, BQ use disorder; SUSRS, Substance Use Severity Rating Scale; YB-OCDRS, Yale-Brown Obsessive Compulsive Disorder Rating Scale; HDRS, Hamilton Depression Rating Scale; BAI, Beck anxiety inventory; BDI, Beck depression inventory.

a Data are expressed in mean ± standard deviation.

### Effect of drug treatment on BQ-CC

Table 3 presents the intention-to-treat proportion, proportion difference and proportion ratio of BQ-CC between different treatment groups. The proportion of BQ-CC in BUD cases who received placebo, escitalopram and moclobemide treatments was 5.4, 34.2 and 33.3%, respectively. Escitalopram and moclobemide groups, respectively, had a 28.7 and 31.2% higher proportion of BQ-CC than that for the placebo group. The ratio of BQ-CC proportion for escitalopram and moclobemide users was found to be 6.3- and 6.8-fold, respectively, as compared to the placebo users.

**Table 3.** Proportion, proportion difference and proportion ratio of betel-quid chewing cessation for 6–8 weeks associated with drug treatment among male betel-quid use disorder chewers

Group	No. of participant	BQ-CC frequency			BQ-CC difference				
		No	Yes	Proportion	Adj. proportion^a^	Adj. PD^a^	(95% CI)	Adj. PR^a^	(95% CI)
Drug treatment									
Placebo	37	35	2	5.4%	5.4%	Ref.		1.0	(Ref.)
Escitalopram	38	25	13	34.2%	34.1%	28.7%	(11.8%–45.6%)	6.3	(1.5–26.1)
Moclobemide	36	24	12	33.3%	36.6%	31.2%	(13.4%–49.1%)	6.8	(1.6–28.0)

BQ-CC, betel-quid chewing cessation; Adj. PD, adjusted proportion difference; Adj. RR, adjusted proportion ratio; Ref., Placebo is the reference group.

a Proportion difference was measured in adjusted risk difference and proportion ratio was measured in adjusted risk ratio obtained from the logistic regression model adjusted for age, educational level, cigarette smoking and the level of betel-quid use disorder.

### Frequency and amount of BQ consumption

**Fig. 1.**
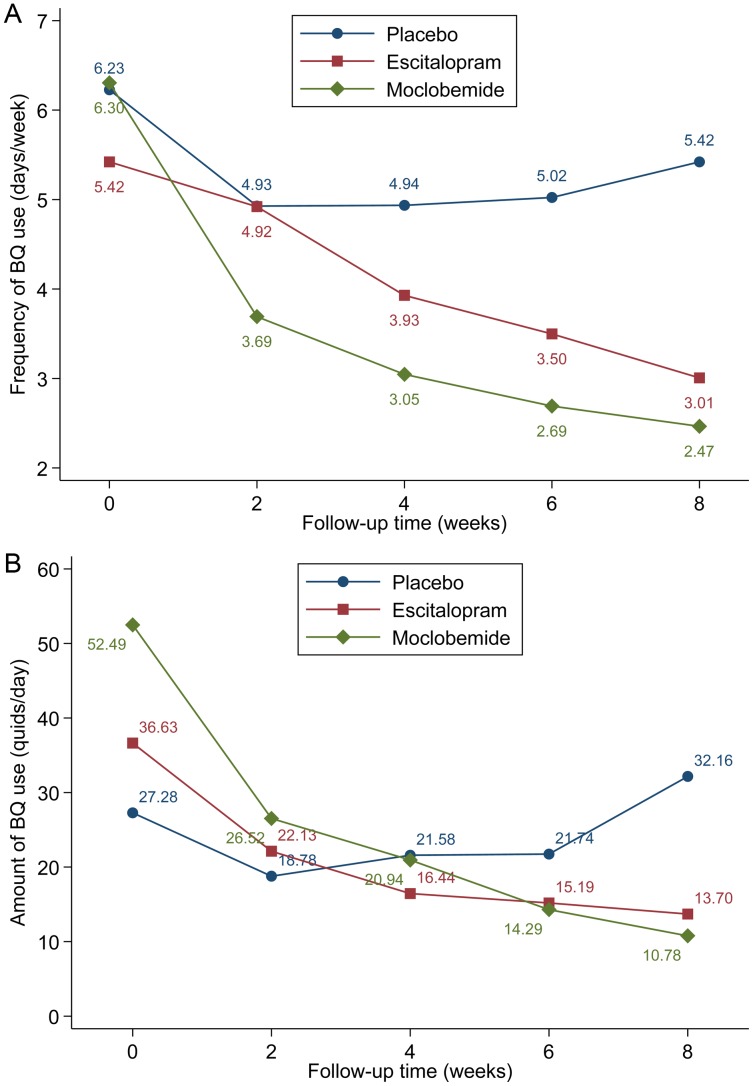
Intention-to-treat cumulative incidences of BQ chewing cessation (BQ-CC) associated with drug treatment groups among male chewers with betel-quid use disorder. **Note**: Cumulative incidences were estimated from the Kaplan–Meier estimators. Log-rank test was used to test the equality of cumulative incidences across drug treatment groups, χ^2^ = 6.640, *p* = 0.036.

Table 4 shows the interaction effects between drug treatments and time on BQ use-associated outcomes. Significant drug treatment and time interactions on the frequency of BQ consumption were found at the 2, 4, 6 and 8 weeks of follow-up, with the lowest frequency observed among BUD cases receiving moclobemide at 8 weeks (model-predicted mean, 2.47 days/week; a 3.76 days/week lower frequency as compared to placebo cases at baseline, 6.23 days/week, Fig. 1*a*). The participants with escitalopram treatment exhibited a significantly lower amount of BQ intake at week 8 (model-predicted mean, 13.70 quids/day; a 13.58 quids/day lower amount as compared to placebo cases at baseline, 27.28 quids/day, Fig. 1*b*). In each 2-week follow-up after the treatment of moclobemide, BUD patients showed a significantly lower amount of BQ intake (all *p* for drug × time interaction ⩽0.006, Table 4). In Figs 1*a* and 1*b*, BUD patients with escitalopram and moclobemide treatments both exhibited a significantly lower frequency and amount of BQ intake at week 8 than those with placebo.

**Table 4.** Main and interaction effects of drug treatments on betel-quid use-associated outcomes over 8 weeks of follow-up

Group	Frequency, days/week			Amount, quids/day			SUSRS, point			YB-OCDRS, point		
	*β*^a^	SE	*p*	*β*^a^	SE	*p*	*β*^a^	SE	*p*	*β*^a^	SE	*p*
*Main effect*												
Drug treatment^b^			**0.300**^c^			**0.002**^c^			**0.943**^c^			**0.887**^c^
Placebo	Ref.^d^			Ref.^d^			Ref.^d^			Ref.^d^		
Escitalopram	−0.81	0.64	0.208	9.35	7.20	0.194	0.55	1.66	0.740	1.59	3.34	0.635
Moclobemide	0.08	0.65	0.906	25.21	7.34	0.001	0.42	1.69	0.804	0.48	3.40	0.888
Time at treatment^b^			**0.032**^c^			**0.093**^c^			**0.062**^c^			**0.103**^c^
0-week (baseline)	Ref.^d^			Ref.^d^			Ref.^d^			Ref.^d^		
2-weeks	−1.30	0.42	0.002	−8.50	4.56	0.063	−2.13	1.09	0.050	−3.53	2.00	0.077
4-weeks	−1.29	0.53	0.015	−5.69	5.85	0.330	−3.99	1.38	0.004	−7.02	2.60	0.007
6-weeks	−1.20	0.60	0.044	−5.54	6.65	0.405	−3.96	1.57	0.011	−6.63	2.98	0.026
8-weeks	−0.81	0.63	0.204	4.88	7.07	0.490	−2.93	1.65	0.076	−6.58	3.20	0.040
Interaction effect (drug × time)			**0.002**^c^			**0.022**^c^			**0.254**^c^			**0.334**^c^
SSRI												
Escitalopram × 2-wk	0.80	0.57	0.161	−6.00	6.26	0.338	0.52	1.49	0.730	−0.38	2.74	0.890
Escitalopram × 4-wk	−0.20	0.73	0.783	−14.49	8.02	0.071	−0.49	1.90	0.797	−3.04	3.56	0.393
Escitalopram × 6-wk	−0.72	0.81	0.377	−15.90	9.03	0.078	−0.07	2.12	0.974	−1.57	4.05	0.698
Escitalopram × 8-wk	−1.61	0.86	0.062	−27.81	9.57	0.004	−2.54	2.24	0.256	−4.32	4.33	0.319
MAOI												
Moclobemide × 2-wk	−1.31	0.58	0.024	−17.47	6.40	0.006	−1.51	1.53	0.322	−3.47	2.81	0.217
Moclobemide × 4-wk	−1.97	0.74	0.008	−25.86	8.17	0.002	−1.30	1.93	0.503	−2.14	3.63	0.556
Moclobemide × 6-wk	−2.41	0.83	0.004	−32.66	9.19	<0.001	−2.33	2.16	0.281	−4.99	4.12	0.226
Moclobemide × 8-wk	−3.03	0.87	0.001	−46.59	9.73	<0.001	−5.49	2.28	0.016	−9.00	4.40	0.041

SUSRS, Substance Use Severity Rating Scale; YB-OCDRS, Yale-Brown Obsessive Compulsive Disorder Rating Scale.

a GEE regression coefficients (*β*) denote age, educational level, cigarette smoking and betel-quid use disorder-adjusted effects of drug treatments and follow-up times on betel-quid use-associated outcomes.

b Placebo is the reference group for drug treatments and baseline is the reference group for follow-up times.

c *p* values for the overall testing of the study explanatory variable or their interaction terms.

d Reference group.

### BQ disorder rating scales

Significant treatment and time interactions on SUSRS and YB-OCDRS for BQ use were observed at week 8 of follow-up, at that time a lower score was reported by moclobemide participants than placebo participants.

### Safety

The adverse events and symptoms occurred in the participants during the 8 weeks of follow-up after drug treatments were shown in online Supplementary Table S1. BUD patients who received placebo, escitalopram and moclobemide treatment had 0, 5.3 and 5.6% of dry mouth, respectively. However, the treatment difference in this event was non-significant. For the psychiatric event, BUD patients with moclobemide (13.9%) showed a significantly higher proportion of dizziness than the other two treatment groups (both 0%). There were no significant differences in liver and renal functions, electrolytes and blood tests before and after 8-week drug treatments were found among the three drug treatment groups.

## Discussion

We demonstrate that prescribing either escitalopram or moclobemide for 6 weeks might be effective in treating BUD patients when compared to the placebo group, in terms of BQ-CC corroborating with significantly reduced frequency and amount of daily BQ consumed. The YB-OCDRS was used to monitor the craving status of BUD, which showed that drugs also decreased the severity in this aspect. Craving is associated with the long-lasting and consolidated memory cued with the substance (Carew and Sutton, 2001; Routtenberg and Rekart, 2005; Holahan and Routtenberg, 2007). It is known that BQ use receives social and cultural cues (Osborne *et al*., 2017), and this study supports that BUD has cue-influenced addictive behaviour and can be alleviated by drug therapy.

MAO is involved in the common degradation pathway for both dopamine and serotonin, thus MAOI prevents the breakdown of neurotransmitters and increases the concentrations of dopamine and serotonin in the brain (Fowler and Tipton, 1984). AN has MAOI-like properties and can inhibit MAOA mRNA and protein expression (Chen *et al*., 2012). In clinics, the use of MAOI, such as moclobemide, is supposed to have a treatment effect on BQ addictive use. In this randomised controlled drug trial, our findings confirmed this hypothesis, in that moclobemide treatment was observed to have a significant effect on BQ chewing abstinence for BUD chewers (adjusted proportion ratio, 6.8 for BQ-CC as compared with placebo).

Escitalopram is an anxiolytic drug and antidepressant by the selective inhibition of the serotonin re-uptake receptor that augments intramural serotonin levels. Its efficacy has been studied in the cocaine and other illicit drugs in mice model (Jastrzebska *et al*., 2017; Smaga *et al*., 2017). In our clinical trial, a significant drug effect of escitalopram on BQ chewing abstinence was identified (adjusted proportion ratio of 6.3 for BQ-CC as compared with placebo). However, the reduced effect of this SSRI on the amount of BQ use was found only at 8 weeks of drug treatment (*p* for interaction effects of escitalopram x 8 weeks is 0.004). This suggests that a significant drug effect of escitalopram on the reduced amount of BQ intake has to treat with at least 8 weeks.

As compared to moclobemide, which is less used in clinical treatment in recent years, escitalopram is a regularly used mild drug with limited clinical complications and is considered a better tolerated antidepressant (Raymond *et al*., 2001). For BQ cessation therapy, escitalopram is recommended to serve as a primary drug for BUD patients, although the treatment effect would be expected before one to two cycles of drug use (approximately 4–8 weeks). For BUD chewers who have a strong quitting willingness or BQ-related oral diseases, moclobemide is still recommended, because a significantly reduced frequency and amount of BQ use was observed at 2 weeks of drug treatment (*p* for interaction effects of moclobemide x 2 weeks on frequency and amount are 0.024 and 0.006, respectively). In addition, these two drugs should not be used in conjunction due to serotonin syndrome.

A few studies reported behavioural (counselling) based cessation programmes for BQ use. A study in dental clinical settings indicated that 32% (71/221) BQ users have a willingness to quit BQ chewing after attending an educational intervention programme; however, no successful quitting rate was reported (Le *et al*., 2014). Four focus groups (24 participants) and 15 depth face-to-face interviews were conducted for BQ current users, in that participants perceived BQ chewing as an addiction and a risk factor for cancer and other health-related conditions, but no cessation case was reported (Lin *et al*., 2017). A behavioural cessation programme conducted in Guam showed that the motivation to stop BQ chewing is difficult to maintain if coworkers or family do not abstain; however, a new type of intervention method for BQ behavioural cessation has been developing (Moss *et al*., 2015). These social and cultural contexts highlight the importance of the development of a pharmacologically assistant therapy for BQ-CC. However, a recent Cochrane systematic review and mete-analysts indicated that individually-delivered counselling for smoking cessation and counselling plus pharmacotherapy can assist smokers to quit (Lancaster and Stead, 2017). Clinical trials of non-pharmacological treatments still are a research direction for BQ cessation in the future.

One patient who received placebo reported the adverse effect of impotency. In this trial, the placebo was produced with artificial capsules with no therapeutic or pharmacological effects by a professional pharmaceutical company. It cannot be confirmed whether this side effect was associated with the use of the non-medical capsule or other drugs, or this is a pre-existing symptom in this patient who did not report it previously. Because we did not find a significant difference in the proportions of this type of event across three treatment groups (online Supplementary Table S1), and only one event was observed. We tend to consider that this event is not related to the use of placebo.

A limitation in our study is the fixed dosage (moclobemide at 150 mg/day and escitalopram at 10 mg/day) and 8-week duration of therapy. Increasing daily dosage and lengthening duration of treatment may be required to investigate the efficacy of drug treatment in patients with different BUD severity levels. Additionally, we observed a higher rate of dropout in the placebo group after this clinical trial has been unbound. This condition may be related to the lack of a treatment effect experienced by this group. Nonetheless, the proportion of completion of assessment at each follow-up and the completion of BQ-CC evaluation were consistently similar across the three drug treatment groups. There are not validity and reliability data for YB-OCDRS used in Chinese populations for BQ addictive use. The derived scores and the evaluation of the severity of craving for BQ use disorder need to be validated in the future study.

## Conclusions

BUD chewers respond to pharmacotherapy. Prescribing a fixed dose of moclobemide and escitalopram to BUD patients over 8 weeks demonstrated treatment benefits to BQ-CC. Future studies can build upon our findings and investigate the correct dosage and duration of administration that correlate with the BUD severity levels. This study is most definitely providing a contribution to new knowledge and may even be pioneering, but as the first study of its kind with a relatively small sample, it only provides preliminary evidence and requires replication in larger trials.

## Supplementary material

For supplementary material accompanying this paper visit http://dx.doi.org/10.1017/S2045796020000384. click here to view supplementary material

## Data

Due to constraints on the data sharing permissions of the samples included in this study, we are not allowed to share the data for public use.
