# Supplementary material for: Effect of antidepressants for cessation therapy in betel-quid use disorder: a randomised, double-blind, placebo-controlled trial
Source: Epidemiol Psychiatr Sci. 2020 May 6;29:e125. doi: 10.1017/S2045796020000384 (PMC7216045; doi:10.1017/S2045796020000384)
Supplement: Supplementary file 1 [file S2045796020000384sup001.pdf]

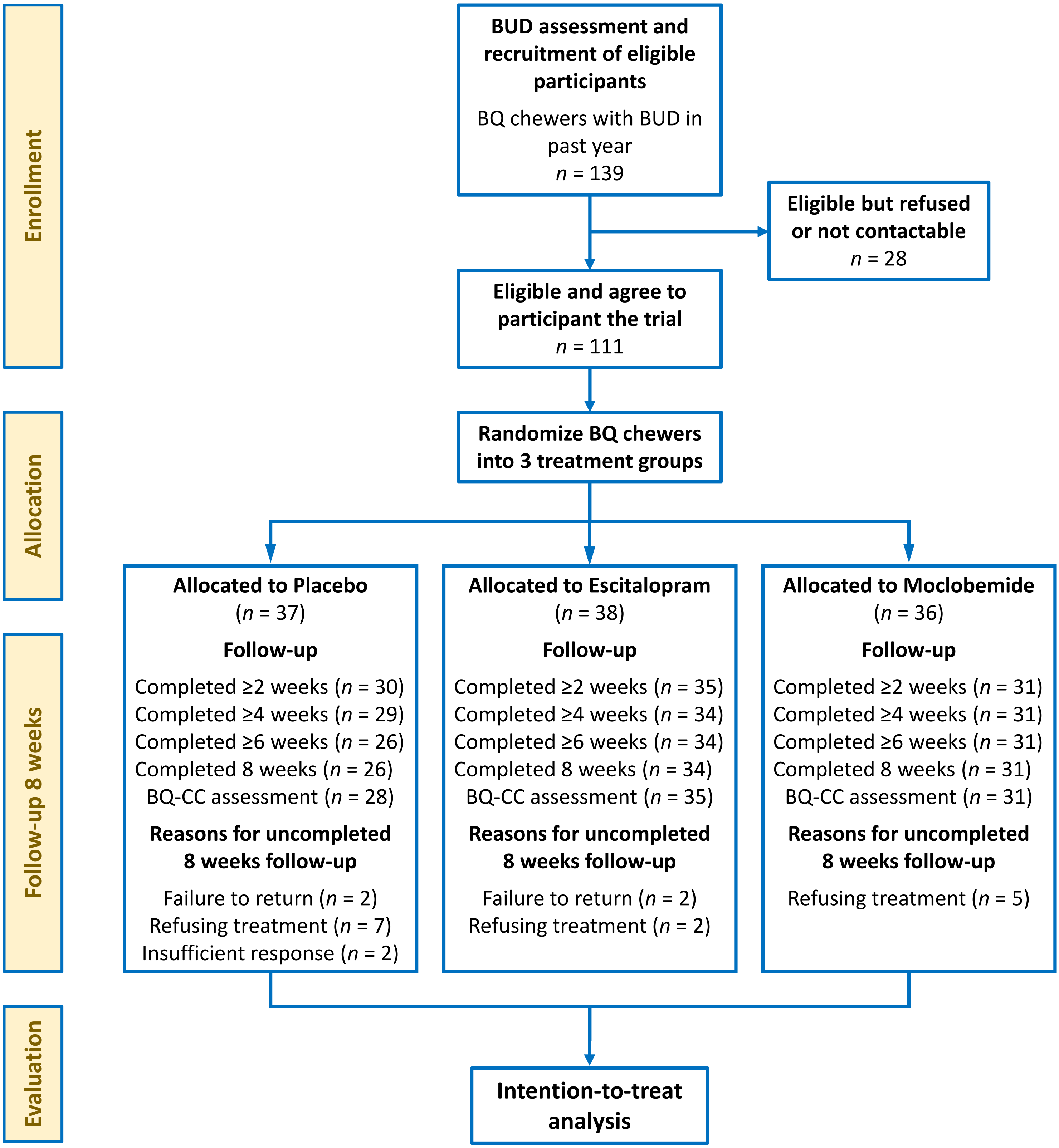

Supplementary Fig 1. CONSORT Diagram for the evaluation of drug treatments for male chewers with betel-quid use disorder: a randomized placebo-controlled clinical trial. **Note**: BQ-CC denotes betel-quid chewing cessation.

Supplementary Table 1. Adverse events occurred during the 8-weeks of follow-up after drug treatments

Events	Placebo (n=37)		Escitalopram (n=38)		Moclobemide (n=36)		*P* value[a]
	No.	(%)	No.	(%)	No.	(%)	
**Gastrointestinal event**							
Constipation	0	0	1	2.6	0	0	1.000
Diarrhea	1	2.7	0	0	0	0	0.658
Nausea	0	0	1	2.6	0	0	1.000
Dry mouth	0	0	2	5.3	2	5.6	0.465
**Psychiatric event**							
Dizziness	0	0	0	0	5	13.9	0.003
Insomnia	1	2.7	0	0	2	5.6	0.210
**Other systems**							
Impotency	1	2.7	0	0	0	0	0.658

[a]*P* values were obtained from the Fisher's exact test.

Supplementary Table 2. The distribution and difference of clinical outcomes between before and after 8-weeks of drug treatments

Factors	Placebo			Escitalopram			Moclobemide		
	Before	After	***P***[a]	Before	After	***P***[a]	Before	After	***P***[a]
	n **= 35**	***n*** **= 22**		***n*** **= 36**	***n*** **= 28**		***n*** **= 34**	***n*** **= 23**	
**Liver function**									
GOT (IU/L)	34.9±18.9	27.1±8.9	0.079	37.6±21.3	31.1±18.9	0.213	40.1±33.0	31.5±17.7	0.256
GPT (IU/L)	39.5±29.6	32.9±22.8	0.372	33.6±25.4	31.0±18.8	0.651	42.0±31.7	33.7±22.8	0.286
**Renal function**									
BUN (mg/dL)	11.9±5.3	13.0±4.4	0.383	11.5±6.5	10.3±3.8	0.407	11.0±5.1	11.4±3.7	0.728
Creatinine (mg/dL)	0.9±0.2	0.9±0.2	0.949	1.0±0.5	0.9±0.2	0.330	1.0±0.2	1.0±0.3	0.360
**Electrolytes**									
Sodium (mmol/L)	138.9±3.8	138.7±2.8	0.891	138.5±3.4	139.4±2.2	0.239	136.7±3.5	137.7±2.7	0.252
Potassium (mmol/L)	4.2±0.4	4.1±0.4	0.184	5.0±5.7	4.0±0.4	0.357	4.0±0.5	4.1±0.4	0.607
**Blood Tests**									
WBC (10^3/ul)	8.1±2.2	1.3±2.5	0.256	8.7±2.5	7.9±1.7	0.161	8.5±3.6	8.6±2.6	0.965
Hemoglobin (g/dL)	15.1±1.9	15.2±1.6	0.881	14.4±2.1	14.7±2.1	0.619	14.4±3.0	15.2±1.4	0.238
Platelets (10^5/ul)	2.3±0.7	2.5±1.3	0.563	2.5±0.9	2.4±0.5	0.529	2.9±1.6	2.8±2.3	0.849

GOT, glutamic oxaloacetic transaminase; GPT, glutamic pyruvic transaminase; BUN, blood urea nitrogen; WBC, white blood cell.
[a]P values for the difference before and after drug treatment.
